# Progression of the “Psychological Typhoon Eye” and Variations Since the Wenchuan Earthquake

**DOI:** 10.1371/journal.pone.0009727

**Published:** 2010-03-17

**Authors:** Shu Li, Li-Lin Rao, Xin-Wen Bai, Rui Zheng, Xiao-Peng Ren, Jin-Zhen Li, Zuo-Jun Wang, Huan Liu, Kan Zhang

**Affiliations:** 1 Institute of Psychology, Chinese Academy of Sciences, Beijing, China; 2 Graduate University of Chinese Academy of Sciences, Beijing, China; Aga Khan University, Pakistan

## Abstract

**Background:**

In 2008 after a massive earthquake jolted Wenchuan, China, we reported an effect that we termed a “Psychological Typhoon Eye”: the closer to the center of the devastated area, the lower the level of concern felt by residents about safety and health. We now report on the progression of this effect and the development of new variations after the quake as well as investigating potential explanations.

**Methodology/Principal Findings:**

We conducted two sequential surveys of 5,216 residents in non-devastated and devastated areas in September-October 2008 and April-May 2009. Respondents were asked five questions to assess their concerns about safety and health. A MANCOVA showed a significant inverse effect of residential devastation level on the estimated number of medical and psychological workers needed, the estimated probability of an epidemic outbreak, and the estimated number of self-protective behaviors needed (Ps<0.001), in spite of the passage of one year. The level of post-earthquake concern decreased significantly with an increase in the residential devastation level. Additionally, we observed two variations in the “Psychological Typhoon Eye” effect, in that the respondents' concern decreased with increasing relational distance between a respondent and victims who had suffered either **physical** or **economic** damage.

**Conclusions/Significance:**

The previously reported effect of a “Psychological Typhoon Eye” remains robust over a 1-year period. We found that the “psychological immunization” theory did not provide a satisfactory explanation for these intriguing results. Our findings may be useful in understanding how people become resilient to threats.

## Introduction

On May 12, 2008, an 8.0-magnitude earthquake jolted Sichuan and Gansu Provinces in China. The death toll in Sichuan Province was 68,712 with an additional 17,921 listed as missing [1]. About 4.45 million people in the province were injured in the quake, of whom 143,367 were hospitalized, including 10,015 sent out of the province for medical treatment [1]. The catastrophic earthquake dramatically heightened people's concern throughout China about safety and health.

We previously reported a “Psychological Typhoon Eye” effect [2] one month after the earthquake: the closer to the center of the devastated area, the lower a resident's concern about safety and health. In order to investigate the robustness of the earlier finding, to understand the progression of this phenomenon, and to test the potential explanations of this effect, we conducted second and third survey waves.

## Methods

### Data Collection and Sample

We conducted the second and third survey waves four months and eleven months, respectively, after the earthquake (the second wave: September to October, 2008; the third wave: April to May, 2009). The study was part of the Emergency Project to Provide Psychological Assistance in Wenchuan Earthquake Areas (No. KKCX1-YW-05) and received approval from the institutional review board of the Institute of Psychology, Chinese Academy of Sciences. Because the protocol was judged as posing only a low risk, the board recommended oral informed consent, which we obtained from the study respondents.

The sampling frame consisted of 1,038 adults living in non-devastated areas (Fujian Province and Beijing City) and 4,178 adults living in devastated areas (Sichuan and Gansu Provinces). Tropical storm Fengshen lashed the very area of Hunan Province where we had initially conducted our first wave survey after we had completed the first wave, but before we could conduct the second wave (http://news.xinhuanet.com/newscenter/2008-06/26/content_8445157.htm). In order to avoid a potential situation in which respondents in Hunan Province saw themselves as disaster victims, we no longer chose Hunan Province (which we had sampled in the first wave of the survey) as a representative sample of a non-devastated area.

Respondents were recruited by going door to door and asking people to participate in the questionnaire. As a result, some respondents participated in all three waves, whereas others participated in some waves, but not others. Entry criteria included an age of at least 18 years, literate, and willing to provide personal contact information. We paid respondents a small fee or gave them a small present such as a bar of soap, a towel, or a packet of washing powder for each completed questionnaire. Table 1 summarizes the respondents' demographic data.

**Table 1:** Demographic characteristics of residents in non-devastated areas and devastated areas.

		Second Survey	Third Survey
Characteristic		Residence in Non-devastated Areas	Residence in Devastated Areas	Residence in Non-devastated Areas	Residence in Devastated Areas
		(N = 392)	(N = 2,099)	(N = 646)	(N = 2,079)
Age (yr)					
	Mean	36.3±14. 9	34.3±9.8	32.7±9.3	34.3±9.9
	Median	30	33	30	34
		No. of Respondents (%)			
Sex					
	Male	181 (46.2)	739 (35.2)	276 (42.7)	704 (33.9)
	Female	204 (52.0)	1337 (63.7)	364 (56.3)	1358 (65.3)
	Unknown	7 (1.8)	23 (1.1)	6 (0.9)	17 (0.8)
Education					
	Below high-school graduate	33 (8.4)	819 (39.0)	26 (4.0)	841 (40.5)
	High-school graduate	66 (16.8)	747 (35.6)	82 (12.7)	651 (31.3)
	Beyond high-school graduate	281 (71.7)	516 (24.6)	526 (81.4)	565 (27.2)
	Unknown	12 (3.1)	17 (0.8)	12 (1.9)	22 (1.1)
Occupation					
	Civil servant	38 (9.7)	39 (1.9)	73 (11.3)	30 (1.4)
	Employee of public institutions	76 (19.4)	173 (8.2)	103 (15.9)	190 (9.1)
	Enterprises employee	148 (37.8)	642 (30.6)	297 (46.0)	744 (35.8)
	Medical worker	17 (4.3)	66 (3.1)	3 (0.5)	67 (3.2)
	Teacher	36 (9.2)	170 (8.1)	56 (8.7)	130 (6.3)
	Farmer	3 (0.8)	432 (20.6)	9 (1.4)	313 (15.1)
	Student	27 (6.9)	21 (1.0)	28 (4.3)	54 (2.6)
	Other	38 (9.7)	496 (23.6)	66 (10.2)	398 (19.1)
	Unknown	9 (2.3)	60 (2.9)	11(1.7)	153 (7.4)

### Key Measures

We used the same questions as in the first survey wave to assess people's concern about safety and health. We asked the respondents in both the devastated and non-devastated areas to indicate the number of relief workers (medical and psychological) that they thought were needed, the probability of an epidemic outbreak, and the amount of self-protective behavior needed. A larger mean would apparently indicate a higher level of concern about safety and health. We also asked only the respondents in the quake area to recommend a dosage of a hypothetical psychological medication for an earthquake victim. The higher the dosage prescribed, the more severe the respondent's perceived trauma. See Table 2 for the details of the five questions. As in the first survey wave [2], we classified the level of residential devastation in the devastated area based on the residents' self-reported assignment of themselves into one of three categories: slightly devastated, moderately devastated, and extremely devastated.

**Table 2:** Post-earthquake concerns about safety and health as assessed from responses to five questions, as related to the respondents' relational distance level.

Question	Wave	Damage	Relational distance	Post hoc tests‡
			Self	Primary	Secondary	Strangers	
			No.	Response	No.	Response	No.	Response	No.	Response	
**1.**	2	Economic	574	16.2±22.9	184	24.1±25.6	526	30.3±30.5	762	32.5±27.0	Self<Primary<Secondary = Strangers
	2	Physical	41	22.9±26.7	157	18.4±31.4	589	19.8±25.5	1242	30.8±27.4	Primary = Secondary<Strangers
	3	Economic	504	24.2±26.1	135	29.6±26.9	522	33.6±34.6	867	39.2±39.4	Self<Secondary<Strangers; Primary<Strangers
	3	Physical	52	26.7±27.5	134	25.4±25.8	468	28.0±34.7	1242	37.7±36.3	Primary = Secondary<Strangers
**2.**	2	Economic	574	26.7±33.1	184	27.0±31.4	526	27.4±33.5	762	37.4±36.7	Self = Primary = Secondary<Strangers
	2	Physical	41	26.9±34.5	157	27.4±34.4	589	25.7±31.5	1242	34.0±35.9	Secondary<Strangers
	3	Economic	504	25.2±30.5	135	27.2±31.7	522	33.5±34.6	867	39.4±36.4	Self<Secondary<Strangers; Primary<Strangers
	3	Physical	52	21.0±27.4	134	21.6±29.9	468	26.5±32.8	1242	38.4±36.0	Primary = Secondary<Strangers
**3.**	2	Economic	574	75.7±169.1	184	95.1±172.9	526	108.2±192.8	762	132.6±215.8	Self<Secondary<Strangers; Primary<Strangers
	2	Physical	41	72.2±158.0	157	117.5±234.5	589	75.5±161.2	1242	121.3±202.7	Primary>Secondary; Secondary<Strangers
	3	Economic	504	68.4±147.9	135	120.9±208.3	522	122.3±202.1	867	117.2±196.2	Self<Primary = Secondary = Strangers
	3	Physical	52	56.6±121.7	134	112.2±215.7	468	111.7±198.8	1242	114.1±191.2	Self = Primary = Secondary = Strangers
**4.**	2	Economic	574	72.3±177.5	184	132.9±238.4	526	146.7±265.9	762	144.5±241.5	Self<Primary = Secondary = Strangers
	2	Physical	41	93.3±215.0	157	118.5±243.0	589	97.2±217.5	1242	136.9±238.1	Secondary<Strangers
	3	Economic	504	76.1±172.1	135	156.9±263.2	522	141.6±243.3	867	130.7±227.2	Self<Primary = Secondary = Strangers
	3	Physical	52	51.4±117.1	134	125.8±236.5	468	123.8±234.5	1242	131.5±228.0	Self = Primary = Secondary = Strangers
**5.** †	2	Economic	564	58.2±35.1	179	63.6±32.9	483	58.7±32.8	501	58.2±32.5	Self<Primary = Secondary = Strangers
	2	Physical	36	54.7±35.0	159	62.3±35.2	567	58.6±34.5	941	58.8±32.5	Self = Primary = Secondary = Strangers
	3	Economic	523	63.0±33.2	127	61.2±33.3	465	56.5±34.2	473	57.7±33.6	Self = Primary; Secondary = Strangers< Self; Primary = Secondary = Strangers
	3	Physical	42	55.8±27.5	138	60.1±35.1	470	58.2±34.5	798	57.9±33.4	Self = Primary = Secondary = Strangers

Notes. The details of the five questions are as follows: 1. What is the probability (0–100%) that an epidemic disease will be widespread in the post-earthquake areas? 2. How many times (out of 100 aftershocks) would residents in earthquake areas need to take safety measures? 3. How many Medical Doctors are needed for every 1000 residents in earthquake areas? 4. How many Psychological Workers are needed for every 1000 residents in earthquake areas? 5. Suppose that there is a medication which can heal the psychological wounds of mass disaster without side effects such as nausea or anaphylaxis. What dose of the medication should be prescribed for an earthquake victim (up to 100 mg daily)?

*Plus–minus values are means ±SD.

† The estimated dosage more than 100 was coded as the maximum value (100 mg daily).

‡ A sign of inequality (<) indicates a statistically significant difference.

Because people react differently to the same stimulus depending on their relational distance [3], [4], we speculated that post-earthquake concern about safety and health would vary with the relational distance between the respondents and victims who had suffered physical and/or economic damage. To determine the relational distance, we asked the respondents to indicate whether they themselves or their relatives had suffered either physical or economic damage in the earthquake. The types of relationships that were evaluated included (a) self, (b) spouse, (c) parents or offspring, (d) siblings, (e) other relatives, (f) acquaintances, (g) strangers, and (h) none. For our data analysis, we classified the relationships into four categories, *i.e.*, self, primary relationships, secondary relationships and strangers. Primary relationships included nuclear family members, *i.e.*, spouse, parents, offspring, and siblings; secondary relationships included other relatives and acquaintances. The most distant category – strangers –consisted of those respondents who had no known relationship with any victims.

Because respondents could select more than one option, we set the relational distance as the closest reported distance. For instance, we classified a respondent into the primary relationship category if he/she reported that his/her parents had suffered economic damage but he/she himself/herself did not, regardless of whether his/her friends suffered economic damage. Using this, we assessed the relationship between people's concern about safety and health and the relational distance to victims who had suffered physical/economic damage.

We attempted to investigate some of the potential explanations discussed in our previous report [2]. To prevent a “second injury” to the victims, we did not assess the extent of the residents' personal exposure to the hazard stimuli until the third survey wave (eleven months after the earthquake). In the third wave, however, we asked respondents to indicate the extent and frequency of their personal exposure to the earthquake damage, using a six-point scale (from “not at all” to “extremely strong” for the extent; from “never” to “always” for the frequency).

## Results

### The robustness of the “Psychological Typhoon Eye” effect

We conducted a multivariate analysis of covariance (MANCOVA) on the four estimates for the number of relief workers (medical doctors and psychological workers) needed, the probability of an epidemic outbreak, and the amount of self-protective behavior needed, using wave (first, second, and third wave) and residential devastation level (extreme, moderate, slight, and non-devastated) as factors, with gender, age, and education as covariates. In order to examine the robustness of the “Psychological Typhoon Eye” effect, we included the data from the first survey wave [2] as a baseline.

The residential devastation level was a significant inverse main effect (Ps<0.001), indicating that concern about safety and health decreased with increased residential devastation level, But we found no significant main effects of wave (Ps≥0.17), except for the estimated probability of an epidemic outbreak (P<0.001). The interactions between wave and residential devastation level were not significant for the estimated numbers of medical doctors and psychological workers needed (P = 0.37 and 0.47, respectively) but were significant for the estimated probability of an epidemic outbreak and for the amount of self-protective behavior needed (both Ps≤0.013).

**Figure 1:**
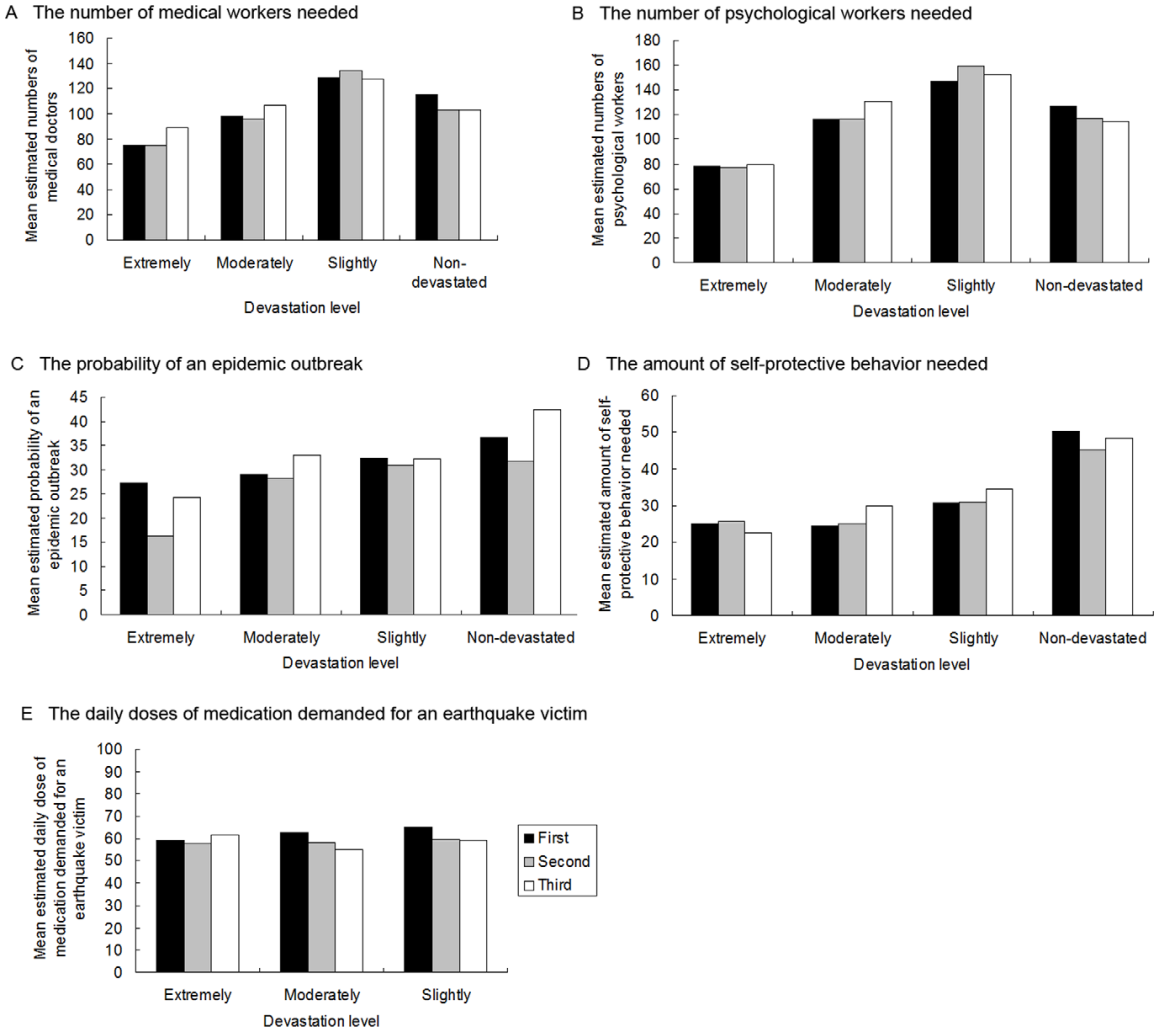
Post-earthquake concerns about safety and health, assessed from responses to five questions, as related to the respondents' residential devastation level.

Least-square difference (LSD) post hoc tests showed that residents in the extremely devastated areas chose the smallest number of relief workers (medical doctors and psychological workers), the lowest probability of an epidemic outbreak, and the smallest amount of self-protective behavior (all Ps<0.001; see Figure 1). The respondents indicated a smaller estimated probability of an epidemic outbreak in the second survey wave (for a mean of 27.2) than in the first and third survey waves (for means of 31.6 and 33.0, respectively; Ps<0.001), with no significant difference between the first and third survey waves (P = 0.10).

An analysis of covariance (ANCOVA) revealed a significant inverse main effect of the residential devastation level on the estimated daily doses of medication needed for an earthquake victim (P = 0.027) and a significant main effect of wave (P = 0.001). The interaction between residential devastation and wave was also significant for the medication estimate (P = 0.004). Residents in the slightly devastated areas indicated a greater daily dose (for a mean of 61.5) than their counterparts in the moderately and extremely devastated areas (means of 59.3, 58.7, respectively; P = 0.023 and 0.035), with no significant difference between the latter two groups (P = 0.67). Respondents indicated a greater daily dose in the first survey wave (for a mean of 62.6) than in the second and third survey waves (for a mean of 58.3, 58.6, respectively; Ps = 0.001), with no significant difference between the second and third survey waves (P = 0.83).

### Variations in the “Psychological Typhoon Eye” effect

To test our speculation about the relational distance (physical and economic), we performed two separate MANCOVAs on the four estimates for the number of relief workers (medical and psychological) needed, the probability of an epidemic outbreak, and the amount of self-protective behavior needed, using wave and relational distance as factors, and gender, age, and education as covariates.

The estimates for the number of relief workers (medical and psychological) needed, the probability of an epidemic outbreak, and the amount of self-protective behavior needed showed a significantly increased, but negative, association with increasing relational distance between respondents and victims who had suffered economic damage (Ps<0.001). We found no significant difference between the second and third survey waves (Ps≥0.18), except for the estimated probability of an epidemic outbreak (P<0.001). The interaction between wave and relational distance was not significant (Ps≥0.09). Our results showed the smallest estimated number of relief workers needed, the lowest probability of an outbreak of an epidemic, and the smallest amount of self-protective behavior needed from respondents who had personally suffered economic damage (Table 2).

We found a similar trend toward a larger estimated number of relief workers needed, a higher probability of an epidemic outbreak, and a larger amount of self-protective behavior needed with an increasing relational distance between the respondents and victims who had suffered physical damage (Ps≤0.022). We did not identify any significant effect of wave (Ps≥0.43), except for the estimated probability of an epidemic outbreak (P = 0.001). The interaction between wave and relational distance was also not significant (Ps≥0.056). Respondents who did not have a relationship with any victims who had suffered physical damage reported the highest estimates on these four issues (Table 2).

The effects of the relational distance between respondents and victims who had suffered either economic or physical damage were not significant with respect to the estimated daily doses of medication needed for an earthquake victim (P = 0.14 and 0.49, respectively, by ANCOVA). There were also no significant differences between the second and third survey waves on the medication estimate (P = 0.89 and 0.79, respectively), and no significant interaction effect (P = 0.06 and 0.96, respectively).

### An alternative explanation for the “Psychological Typhoon Eye” effect

To test whether the “psychological immunization” theory could account for the “Psychological Typhoon Eye” effect, we conducted a MANCOVA on the four estimates for the number of relief workers (medical and psychological) needed, the probability of an epidemic outbreak, and the amount of self-protective behavior needed, using residential devastation level as a factor and with the extent and frequency of personal exposure to the earthquake damage and demographic variables (gender, age, and education) as covariates.

The results revealed that the main effect of residential devastation was significant and inverse (Ps<0.001), except when analyzed against the estimated number of medical workers needed (P = 0.11), indicating that controlling for the extent and frequency of personal exposure to the earthquake damage did not eliminate the residential devastation effect. We found no significant differences for the extent and frequency of personal exposure to the earthquake damage (Ps≥0.32), except that we observed a significant inverse effect of the frequency of personal exposure to the earthquake damage on the estimated number of psychological workers needed (P = 0.03).

An analysis of the estimated daily doses of medication revealed that the residential devastation effect remained significant after controlling for the extent and frequency of personal exposure to earthquake damage (P = 0.013, by ANCOVA). The effect of the extent of personal exposure to the earthquake damage was significant (P = 0.004), while the effect of the frequency of personal exposure was not (P = 0.57).

## Discussion

Based on the results from the three survey waves, we found that the “Psychological Typhoon Eye” effect was robust throughout an entire year. Analyses with demographic variables as covariates did not change the results of the primary analyses that had examined the residential devastation effect. Respondents' concern about safety and health decreased as the residential devastation level increased.

Additionally, we observed two variations in the “Psychological Typhoon Eye” effect from our analysis of the newly collected data. We dubbed these two variations as “guanxi” (relational) versions of the Psychological Typhoon Eye: the closer the relationship between a respondent and victims who had suffered either physical or economic damage, the less the concern about safety and health felt by a respondent. These “guanxi” versions provide mounting evidence to suggest that the degree of an individuals' concern about safety and health did not grow with an increase in the devastation level as we had expected. No one can doubt that the people who were most ravaged by the earthquake were those who lived in the most extremely devastated areas and/or those who themselves suffered the most physical/economic damage. But it seems as though they may be more resilient to disaster, possibly as a result of cognitive dissonance [2], [5].

An earthquake clearly poses a problem as to how to prepare for risk in the face of uncertainty [6]. Surprisingly, our surveys indicate that the strongest resilience to the hazard is achieved by those who reside in extremely devastated areas and those who themselves suffered economic and/or physical damage.

In our previous report^2^, we discussed two possible explanations for this effect, the “psychological immunization” theory [7] and Festinger's theory of cognitive dissonance [5]. The covariance analysis in this current study revealed that the “Psychological Typhoon Eye” effect was independent of the extent of exposure to hazardous stimuli. This evidence leads us to suspect that the “psychological immunization” theory is insufficient to account for the “Psychological Typhoon Eye” effect. Residents were not given an increased psychological immunity to the severe disaster by a personal exposure to hazardous stimuli. As it is difficult to manipulate levels of cognitive dissonance in a field study, a test of the applicability of Festinger's theory of cognitive dissonance to situations such as this will have to be left for future laboratory studies.

We should note that no reports of major epidemics in the earthquake area have surfaced in the 15 months since the earthquake as of the time we prepared this paper. But surprisingly, we observed the highest estimated probability of an epidemic outbreak in the third survey wave. The unexpected rebound in the estimated probability might possibly be due to the fact that China was then on alert to prevent a spread of the 2009 H1N1 infection [8] and that a suspected case of this A/H1N1 flu had been reported at that time in Sichuan, where pig production is most concentrated [9].

We also found a significantly smaller estimated daily dose of medication needed in the second and third survey waves than in the first survey wave. Respondents believed that residents in the devastated areas who had suffered psychological trauma had healed with time. This finding suggests that psychological services have a crucial role in the initial response to massive earthquakes and that time appears to play a protective role in psychological adjustment, at least in people's perceptions.

Although we observed a discrepancy between the devastated and non-devastated areas, we are not suggesting discarding psychological assistance to devastated areas. In all three survey waves the demand for psychological workers was consistently higher than that for medical workers. Psychological workers and policy makers may wish to examine problems from various perspectives to ensure that psychological services are appropriate in areas with different levels of devastation. An awareness of the discrepancy between people inside an area and those from outside the area may enhance judgments in emergency situations and enrich health policy.

The inverse relationship that we found between the devastation level and individuals' concern about safety and health points to a need for further studies to determine whether increasing devastation can increase individuals resilience to different kinds of threats. When preparing for risk in the face of uncertainty, our findings may be useful for understanding how people can become resilient to different kinds of threats. Such investigations have the potential to help people weather damages from nature, disease, or any of the many other threats present on this dangerous planet.
